# Numerical study on solar photovoltaic/thermal system with tesla valve

**DOI:** 10.1038/s41598-024-61785-x

**Published:** 2024-05-12

**Authors:** Shuai Du, Jianxin Zou, Xinli Zheng, Xin Ye, Huazheng Yang, Ping Zhong

**Affiliations:** College of Science, Donghua University, Shanghai, 201620 China

**Keywords:** PV/T, Tesla valve, Heat transfer, Pressure drop, Thermal conductivity, Energy science and technology, Renewable energy

## Abstract

In recent years, photovoltaic/thermal (PV/T) systems have played a crucial role in reducing energy consumption and environmental degradation, nonetheless, the low energy conversion efficiency presents a considerable obstacle for PV/T systems. Therefore, improving heat conversion efficiency is essential to enhance energy efficiency. In this paper, the PV/T system with the Tesla valve is proposed to solve this problem. Firstly, the cooling effect is simulated and analyzed in the system with four different flow channel structures: semicircle, rectangle, triangle and Tesla valve. The results indicate that the system with the Tesla valve exhibits superior cooling performance. Subsequently, several factors including angle, valve number, valve type, and pipe diameter ratio for the Tesla valve are further studied through numerical and simulation analysis. The results reveal that Tesla valves demonstrate optimal cooling performance when possessing the following structural parameters: complete symmetry, more valves, a 30-degree angle and a pipe diameter ratio of 1. Finally, four different types of fluid are selected to explore the Tesla valve. The conclusion shows that nanofluids with high density, low specific heat, and high thermal conductivity also improve the cooling performance. Thus, the PV/T system with the Tesla valve exhibits good heat dissipation and energy storage efficiency, electrical efficiency can reach 16.32% and thermal efficiency reach 59.65%.

## Introduction

Currently, fossil fuels are still the primary source of global energy consumption, comprising approximately 80% of the total global energy consumption^1^. Although fossil fuels play a crucial role in economic growth, social progress, and technological innovation, they lead to environmental pollution and even threaten people's health. According to the International Energy Agency (IEA)^2^, Global CO_2_ emissions from energy combustion and industrial processes are 36.8 Gt in 2022. Therefore, it is quite beneficial to transition towards cleaner energy and improve energy efficiency. Solar energy presents a sustainable, low-cost, clean alternative compared to traditional fossil fuels^3^. With the development of technology, solar energy technology enjoys widespread popularity.

Photovoltaic (PV) technology, representing solar power generation, has reached an advanced stage of maturity^4^. Recent research shows that the maximum photoelectric transform efficiency can reach up to 31%^5^ by using silicon solar photovoltaic cells. However, the photoelectric transformation efficiency of photovoltaic cells cannot surpass 20% under standard conditions. The remaining energy is either absorbed by the photovoltaic cells or dissipated, resulting in significant energy loss. Additionally, a rise of 1 °C in surface temperature leads to a corresponding reduction in efficiency by 0.5%. Consequently, temperature emerges as a critical factor limiting the development of photovoltaic technology.

To address these challenges, researchers have combined photovoltaic cells with solar collector thermal technology. In the system, fluid within the thermal collector removes the thermal energy from the cells by cooling the PV. Thus, further research is focused on improving heat transfer and energy efficiency through variations in structure and fluid selection.

The cooling system uses fluid to realize the thermal energy transfer between PV panels and pipes while promoting heat dissipation and improving electric conversion efficiency^6^. The typical media include air^7,8^, water^9,10^ and nanofluids^11–13^. Water or air-based PV/T cooling systems offer low costs and allow for balanced energy distribution. However, the system is prone to freezing in low-temperature conditions, and there is still room for improvement in energy conversion efficiency. In contrast, nanofluid-based PV/T cooling systems exhibit higher heat transfer efficiency and can be transmitted through microchannels, which are not easy to freeze. Nonetheless, their higher cost remains a drawback.

Kern and Russell^14^ proposed solar photovoltaic solar thermal (PV/T) systems in 1978, and the technology was validated by experimental data using fluids such as air or water as the cooling medium. Chowdhury^15^ discussed the progress of PV/T systems cooled by air, water, and nanofluids and showed that PV/T systems would play a significant role in developing future renewable technologies. Kumar^16^ analyzed the PV/T air system and found that it produced a more substantial energy yield per unit area of the collector than a standalone battery or thermal system. Sarhaddi^17^ investigated the thermal and electrical performance of PV/T air collectors and revealed that increased inlet velocity and solar radiation intensity led to increased energy efficiency and thermal efficiency in the system. A Shahsavar^18^ studied the different PV/T air collector types with and without the cover plate. The PV/T system utilized thin metal sheets that improved heat extraction from PV panels, resulting in effective thermal and electrical efficiency. Fterich Mohamed^19^ analyzed heat transfer in a PV/T air collector prototype using numerical simulation and experimental study. The results indicated that with the best thermal efficiency of 42.5%, the temperature of the PV cell reached a maximum of 75 °C at an airflow rate of 0.5 m/s and 59 °C at an airflow rate of 2 m/s, with a maximum temperature difference of 20 °C between the two experiments. Ramdani Hamza^20^ presented a novel water-based hybrid PV/T collector design that utilized a layer of water to cool PV cells and filter incoming solar radiation. The study found that the proposed design outperforms previously proposed ones in terms of energy efficiency. Hassan Atazaz^21^ analyzed the influence of different parameters on PV/T water.

In recent years, nanofluids have received widespread attention owing to their outstanding thermal conductivity^22–24^. Studies have shown that nanofluids used in cooling PV/T systems can significantly enhance performance, as well as thermal and electrical efficiency. Currently, most research employs common nanofluids, such as Al_2_O_3_ and CuO. Sharaf^25^ analyzed the PV/T system using nanofluids. The results indicated that Al_2_O_3_H_2_O nanofluids were more advantageous than Al_2_O_3_ nanofluids in terms of thermal properties. Michael^26^ performed experiments on a rectangular PV/T system using CuO nanofluids and found that the system's thermal performance was significantly enhanced. Venkatesh^27^ utilized water-based graphene nanofluids as the medium for heat transfer in the PV/T system. The results showed that the graphene nanofluid significantly reduced the PV panel temperature and improved the photovoltaic efficiency and overall efficiency.

The above discusses the influence of fluid on PV/T systems, however, the structural parameters of the flow channel can also have an important impact on PV/T systems. Shen^28^ designed a pipe with a shark dorsal fin type based on the theory of field synergy between velocity and temperature, thereby enhancing the heat transfer performance of the fluid. Kianifard^29^ designed a PV/T collector cooled by water using a serpentine semicircular pipe. The pipe connected the water directly to the heat-absorb plate. Singgih Dwi Prasetyo^30^ analyzed the electrical and thermal efficiency of the PV/T system by exploring different levels of radiation intensity, collector design, fluid type, and flow rate.

With the development of technology, the Tesla valve structure has attracted attention as a new fluid pipeline structure type^31–33^. Tesla valves can enhance heat transfer by creating vortices and turbulence in the fluid flow, which increases the heat transfer coefficient and reduces the temperature difference between the fluid and the solid surface. Some researchers have utilized numerical simulations or experiments to optimize the geometry, number of stages, angles, and tube diameters of Tesla valves, and improve the heat transfer performance. Jingyuan Qian^34^ conducted a numerical study on the Al_2_O_3_-water nanofluid based on the Tesla valve. Bohua Sun^35^ proposed the Tesla valve with a symmetric structure and analyzed fluid flow characteristics in the system using simulation and dimensional analysis. Wang^36^proposed the three-dimensional Tesla valve-typed structure and explored optimizing parameters using an annealing algorithm. Yiwei Fan^37^ proposed a battery thermal management system combining Multi-stage tesla valve liquid cooling and Phase change material. All of the above research applies the Tesla valve structure in heat transfer or heat dissipation with satisfactory feedback. However, the optimal design parameters for Tesla valves under different flow conditions and objectives still require additional exploration.

Thus, this paper aims to contribute by simulating and analyzing the PV/T system with four different flow channel structures and further investigating the optimal design parameters for Tesla valves. This study not only enhances the thermoelectric performance of the PV/T system but also serves as a reference for exploring research on Tesla valves in the field of heat dissipation.

All the parameters are explained in this section, as shown in Table 1.

**Table 1:** Description of symbols.

Symbols	Description	Values
$v_{w}$	Water kinematic viscosity	m^2^/s
$$u_{x}$$$$u_{y}$$$$u_{z}$$	Velocity component	m/s
$p$	Water pressure	bar
$c_{pw}$	Fluid-specific heat capacity	J/(kg K)
$T$	Temperature	K
$k_{w}$	Fluid heat transfer coefficient	/
$S_{T}$	The internal heat source of the fluid	/
$\eta_{all}$	Total efficiency	%
$\eta_{pv}$	Electrical efficiency	%
$\eta_{a}$	Thermal efficiency	%
$\zeta$	Filling factor of the PV panel	/
$\eta_{power}$	The conversion factor of thermal energy to electrical energy	38%
$\eta_{ref}$	The PV panel efficiency under standard test conditions	16.5%
$B_{r}$	Temperature coefficient	0.0045 K^−1^
$T_{pv}$	PV cell operating temperature	K
$T_{ref}$	Battery temperature under standard test conditions	298.15 K
$m$	Fluid mass flow rate	kg/s
$T_{out}$	Temperature of outlet	K
$T_{in}$	Temperature of inlet	K
$A_{c}$	The effective area of heat collection	m^2^
$G$	Solar irradiance	W/m^2^
$Re$	Reynolds number	/
$\rho$	Fluid density	kg/m^3^
$V$	Fluid velocity	m/s
$D$	The hydraulic diameter of the channel	m
$\mu$	Dynamic viscosity	N s/m^2^
$A$	Cross-sectional area of the channel	m^2^
$P$	The perimeter of the inlet	m
$P_{r}$	Reverse flow pressure	bar
$P_{f}$	Forward flow pressure	bar
$D_{i}$	Pressure drop ratio	m/s
$\Delta P_{r}$	The reverse flow pressure difference between the valve outlet and inlet	bar
$\Delta P_{f}$	The forward flow pressure difference between the barrel valve outlet and inlet	bar
$Q_{f}$	Flow rate for forward flow of liquids	L/s
$Q_{r}$	Flow rate for the reverse flow of liquids	L/s
$E$	Throttling efficiency	%
$\Delta P$	Pressure drop	bar
$L$	The pipe length	m
$\lambda$	The resistance coefficient	/
$L_{pv}$	The PV panel length	m
$f_{d}$	The distance between valves	m
$N$	Valve number	pcs
$l$	The tesla valve length	m
$x$	The inlet and outlet length	m
$\mathop T\limits^{-\!\!-}_{{{\text{cell}}}}$	The PV average temperature	K
$a$,$k$	Constant	/
$N_{\max }$	The maximum valve number	pcs

## Numerical analysis

### Physical model design

**Figure 1:**
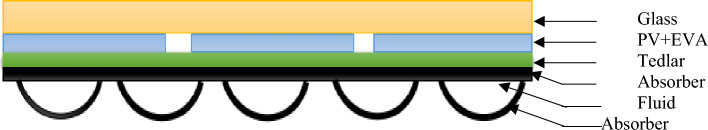
Schematic diagram of the PV/T system structure.

The study focuses on designing a solar PV/T system^38^, which includes glass, PV cells, a heat-absorbing plate, a flow channel, fluid, ethyl vinyl acetate (EVA) and TPT. The system is illustrated in Fig. 1.

**Figure 2:**
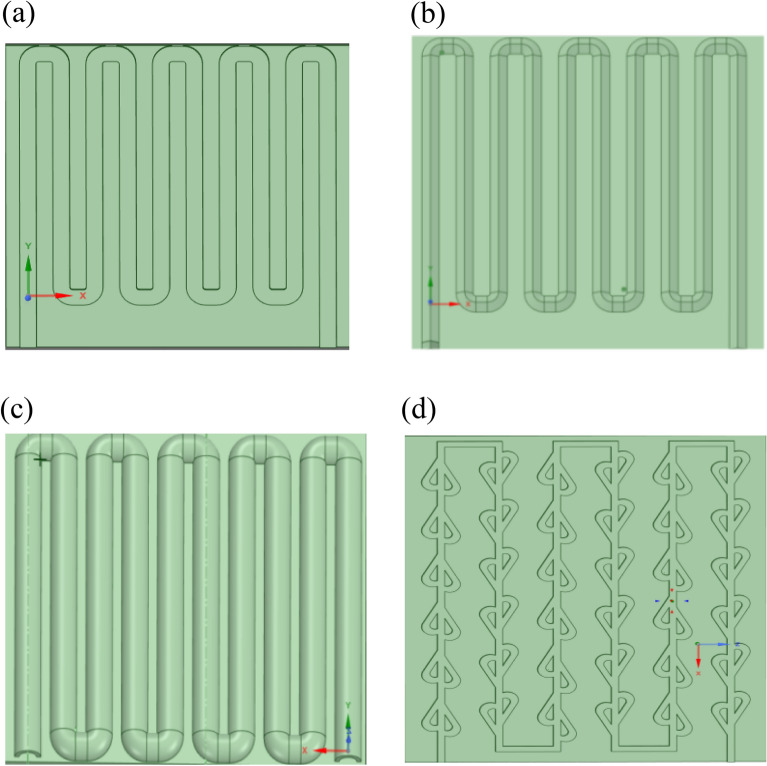
Schematic diagrams of different shapes of runner structures: (**a**) rectangle; (**b**) triangle; (**c**) semi-circle; (**d**) tesla valve.

Several metrics for the colding plate of circular, rectangular, triangular and Tesla valve channel types are shown in Fig. 2.

The model parameters of the four runner structures are shown Table 2.

**Table 2:** Parameters of photovoltaic panels and four runner structures.

Type	Long/diameter (cm)	Wide/radius (cm)	Square (cm^2^)
Semi-circle	3	1.5	3.54
Rectangle	3	1.18	3.54
Triangle	3	2.36	3.54
Tesla valve	1.18	3	3.54
PV mode	61	54	3294

### Model assumptions

The following premise and hypothesis are used:

- The study assumes that only direct solar radiation reaches the surface of the collector. Radiations from the sky and glass cover are neglected.
- Radiations between the cover plate and the heat-absorbing plate are neglected.
- One-dimensional heat transfer takes place vertically through the PV panel.
- The fluid within the airflow channel or water pipe is considered to be an incompressible Newtonian fluid.
- Heat dissipation from the collector and bottom surface is not taken into consideration.
- The fluid motion within the system is modeled as three-dimensional.

### Control equation

The fluid is numerically calculated based on the flow equations governing mass conservation, energy conservation, and momentum conservation. The steady-state continuity^20^ equation is expressed by Eq. (1):

1 $$ \frac{{\partial u_{x} }}{\partial x} + \frac{{\partial u_{y} }}{\partial y} + \frac{{\partial u_{z} }}{\partial z} = 0 $$

The momentum conservation equation is expressed by Eq. (2):

2 $$ \begin{gathered} u_{x} \frac{{\partial u_{x} }}{\partial x} + u_{y} \frac{{\partial u_{x} }}{\partial y} + u_{z} \frac{{\partial u_{x} }}{\partial z} = - \frac{1}{{\rho_{w} }}\frac{\partial p}{{\partial x}} + v_{w} \left( {\frac{{\partial^{2} u_{x} }}{{\partial x^{2} }} + \frac{{\partial^{2} u_{x} }}{{\partial y^{2} }} + \frac{{\partial^{2} u_{x} }}{{\partial z^{2} }}} \right) \hfill \\ u_{x} \frac{{\partial u_{y} }}{\partial x} + u_{y} \frac{{\partial u_{y} }}{\partial y} + u_{z} \frac{{\partial u_{y} }}{\partial z} = - \frac{1}{{\rho_{w} }}\frac{\partial p}{{\partial y}} + v_{w} \left( {\frac{{\partial^{2} u_{y} }}{{\partial x^{2} }} + \frac{{\partial^{2} u_{y} }}{{\partial y^{2} }} + \frac{{\partial^{2} u_{y} }}{{\partial z^{2} }}} \right) \hfill \\ u_{x} \frac{{\partial u_{z} }}{\partial x} + u_{y} \frac{{\partial u_{z} }}{\partial y} + u_{z} \frac{{\partial u_{z} }}{\partial z} = - \frac{1}{{\rho_{w} }}\frac{\partial p}{{\partial z}} + v_{w} \left( {\frac{{\partial^{2} u_{z} }}{{\partial x^{2} }} + \frac{{\partial^{2} u_{z} }}{{\partial y^{2} }} + \frac{{\partial^{2} u_{z} }}{{\partial z^{2} }}} \right) \hfill \\ \end{gathered} $$

The energy conservation equation is expressed by Eq. (3):

3 $$ \rho_{w} c_{pw} \cdot \left( {u_{x} \cdot \frac{\partial T}{{\partial x}} + u_{y} \frac{\partial T}{{\partial y}} + u_{z} \frac{\partial T}{{\partial z}}} \right) = k_{w} \left[ {\frac{{\partial^{2} T}}{{\partial x^{2} }} + \frac{{\partial^{2} T}}{{\partial y^{2} }} + \frac{{\partial^{2} T}}{{\partial z^{2} }}} \right] + S_{T} $$

### Meshing strategy

**Figure 3:**
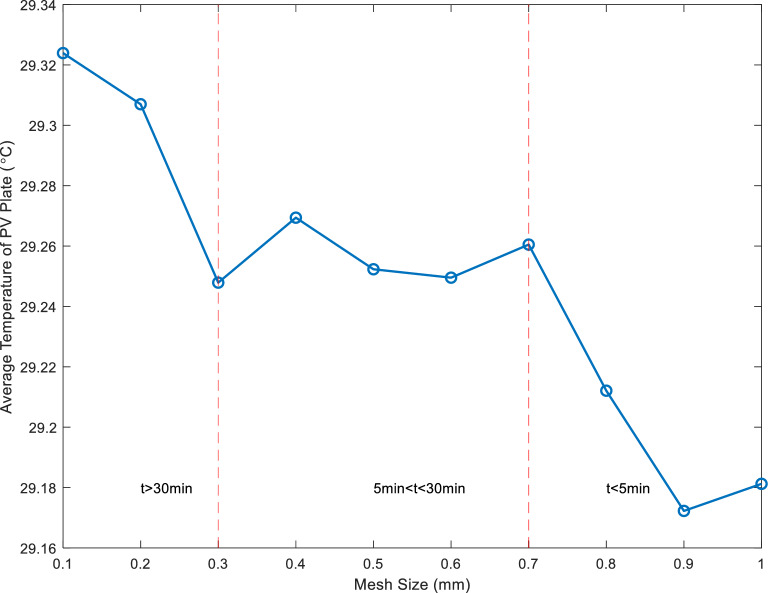
Grid independence verification.

The mesh size significantly influences simulation results. Refining the mesh brings simulation results closer to reality, but it also significantly increases the computational burden. Therefore, conducting mesh independence validation is essential for determining an appropriate mesh size. In this article, grid sizes ranging from 0.1 to 1 mm were selected, with temperature and chosen time factors as verification conditions to assess the suitability of the mesh size. The Tesla valve structure was employed for mesh-independent verification. The result is shown in Fig. 3.

Figure 3 demonstrates that using the grid element size between 0.3 and 0.7 mm yields get more stable results without requiring excessive simulation time, therefore, the mesh element size of 0.5 mm was selected for simulations in this study.

### Numerical solution

ANSYS Student 2024 R1 (https://www.ansys.com/academic/students/ansys-student) software was used for the thermal simulation. ANSYS Student 2024 R1 was separated by finite volume algorithm and second order upwind scheme was chosen to momentum, continuity and energy equations. During the computation, residual parameters accurate to 10^–6^. SIMPLE algorithm was chosen to discretize Control equations. To make the simulation results more accurate, the DO radiation model is used, which has the property of solving the radiation problem for all optical depth ranges.

### Temperature cloud map

**Figure 4:**
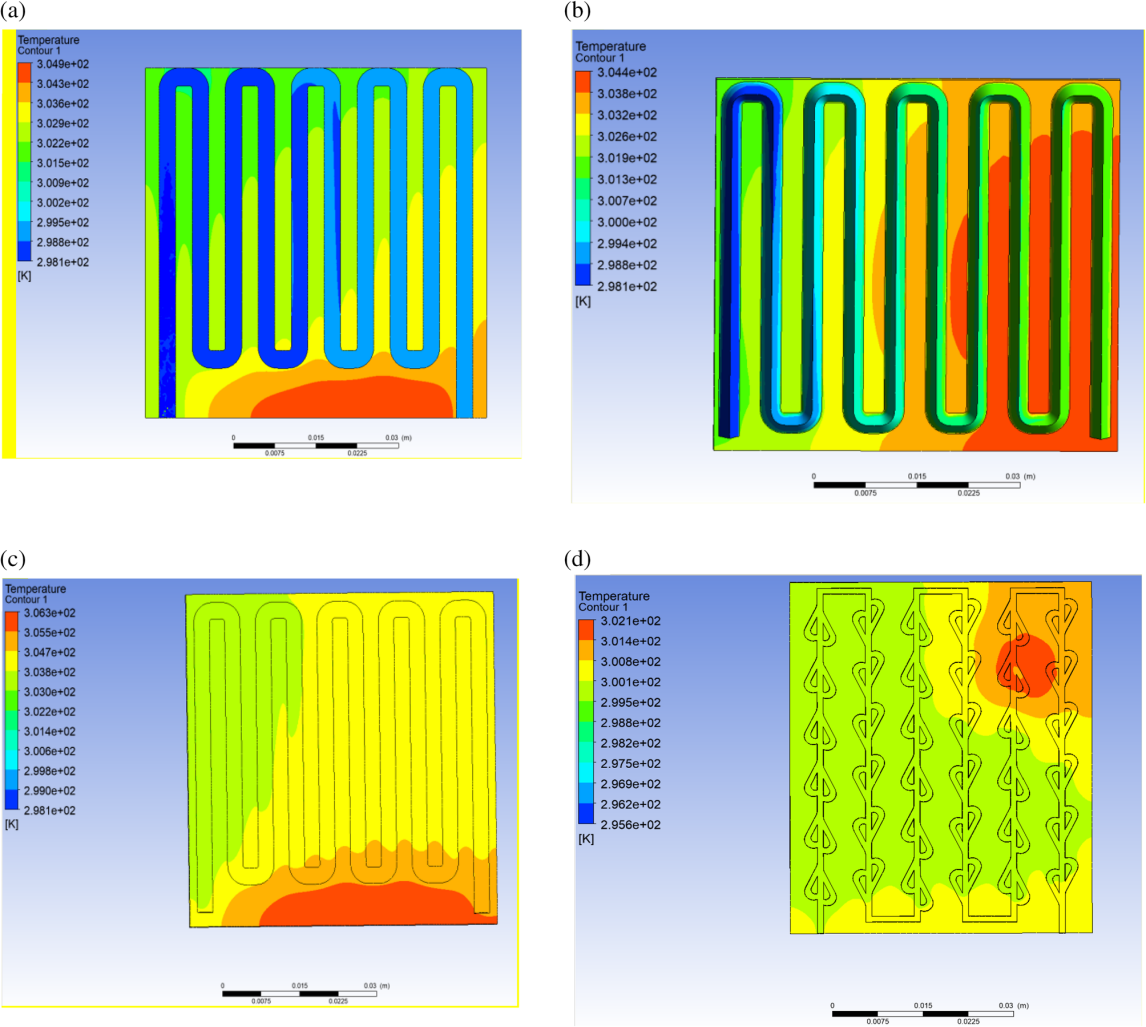
Temperature cloud map with different runner structures: (**a**) rectangle; (**b**) triangle; (**c**) semi-circle; (**d**) tesla valve.

The panel temperature distribution cloud diagrams under four different runner structures were explored through simulation, as shown below in Fig. 4:

Temperature cloud maps reflect the temperature distribution of the panels. As observed from the temperature cloud map, the cooling effect of the PV/T with Tesla valve structure on the panels is more ideal compared to other runner structures.

### Evaluation performance

The system performance is mainly composed of electrical and thermal efficiency^38^; the performance evaluation is expressed by Eq. (4):

4 $$ \eta_{all} { = }\eta_{a} { + }\zeta \frac{{\eta_{pv} }}{{\eta_{power} }} $$

The electrical efficiency is expressed by Eq. (5):

5 $$ \eta_{pv} = \eta_{ref} [1 - B_{r} (T_{pv} - T_{ref} )] $$

The thermal efficiency is expressed by Eq. (6):

6 $$ \eta_{a} = \frac{{c_{pw} \cdot m \cdot (T_{out} - T_{in} )}}{{G \cdot A_{c} }} $$

## Analysis of results

Simulation analysis is conducted on the PV/T system with four different structures. The following parameters are utilized for the simulation: heat flux is 800 W/m^2^, atmospheric temperature is 298.15 K, convective heat transfer coefficient of 10W/m^2^K, and fluid medium is water with a temperature of 298.15 K. Then, the Re is calculated by Eq. (7):

7 $$ Re = \frac{\rho VD}{\mu },\,\,\,D = \frac{4A}{P} $$

8 $$ {\text{Then}}:\,\,\,{\text{Re}} = \frac{4\rho VA}{{\mu P}} $$

Reynolds number (Re)^35^ is related to the viscosity, flow velocity, and the flow channel area. Keeping the tube diameter constant while the sufficiently high flow rate creates turbulence and then can enhance the cooling effect. Therefore, the k-ε model is adopted.

### Cooling effects of four structures

**Figure 5:**
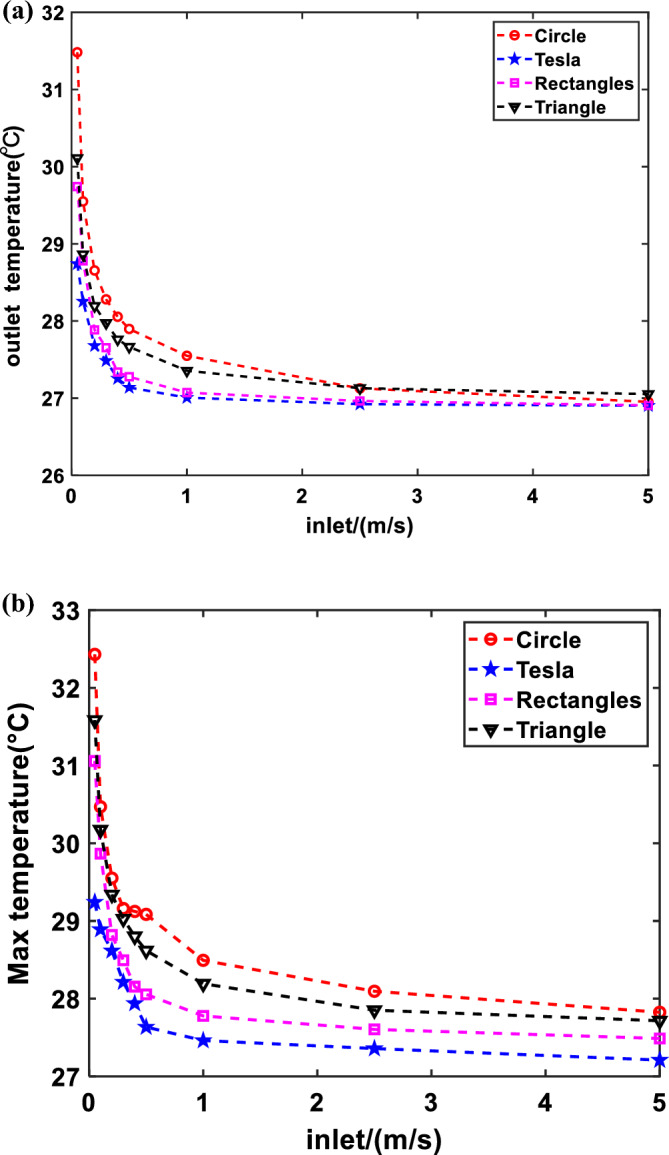
Relationship between fluid flow velocity and temperature for different flow channel structures: (**a**) fluid flow velocity and outlet fluid temperature; (**b**) fluid flow velocity and cell temperature.

The cooling effect of the Tesla valve is investigated by comparing it to the cooling effects of three different cross-sectional structures. Figure 5 below displays the comparing results.

As illustrated in Fig. 5, the cooling effect will be significantly affected by the speed increase. In particular, when the velocity lies within the range of the turbulent critical value to 1 m/s, the increase in flow velocity has a significant effect on lowering the PV temperature. However, when the flow velocity exceeded 1 m/s, there was no significant cooling effect with the increase in flow rate. When warm water is required, the fluid must be kept in the pipeline for a longer amount of time to allow for full heat exchange. This is accomplished by lowering the flow rate (laminar flow state) to create a more efficient heat source.

Indeed, increasing the water flow velocity reduces both the water and PV temperatures. At the same time, the outlet temperature decreases gradually with increasing velocity. As the fluid inlet flow rate increases, the time required for the fluid in the pipe to reach a turbulent state decreases. Consequently, the fluid absorbs less heat energy, leading to a lower outlet temperature. As with the outlet water temperature, the temperature of the PV decreases when velocity increases. The increase in fluid velocity causes a large amount of heat to be taken away as the fluid flows through the PV. While the fluid outlet temperature is decreasing per unit of time, the amount of water flowing out increases.

According to Fig. 5, it is evident that the outlet temperature of the flow channel structure of the Tesla valve is lower compared to the other three flow channel configurations. The superior performance of the forward Tesla valve can be attributed to the acceleration principle, which results in a higher exit velocity compared to the inlet velocity. When the inlet flow rate is constant, the fluid spends less time in the piping of the Tesla valve structure compared to other configurations, outlet temperature is even lower. Conversely, the flow rate through the Tesla valve pipe is greater during the same time, effectively removing more heat from the surface of the PV plate. This further substantiates the superior cooling effect of the Tesla valve structure.

The cooling effect is stable when the velocity exceeds 1 m/s, the outlet temperature and the PV panel temperature change slowly, and the thermoelectric efficiency is not significantly affected. Therefore, it is essential to prioritize the investigation of flow rates below 1 m/s when assessing thermoelectric efficiency.

**Figure 6:**
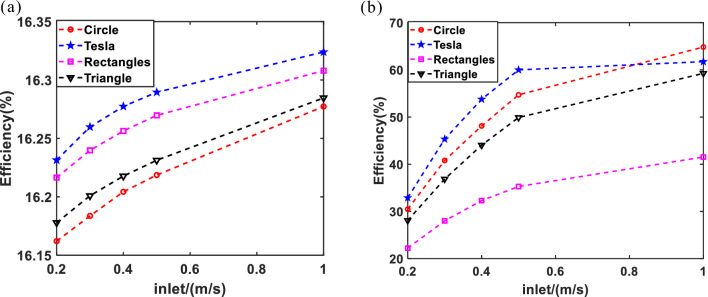
The thermoelectric efficiency of the four structures: (**a**) electrical efficiency; (**b**) thermal efficiency.

It is necessary to evaluate the thermoelectric efficiency of the four structures. As shown in Fig. 6.

The cooling effect of the Tesla valve structure and thermoelectric efficiency have been analyzed based on Fig. 6. The results indicate that the cooling efficiency demonstrated by the Tesla valve is significantly higher, with electrical efficiency reaching 16.32% and thermal efficiency reaching 59.65%. However, various factors such as the angle, number, type, and diameter of the valve influence the cooling efficiency. Therefore, additional experimental studies are warranted.

### Analysis of Tesla valve

#### Principle

**Figure 7:**
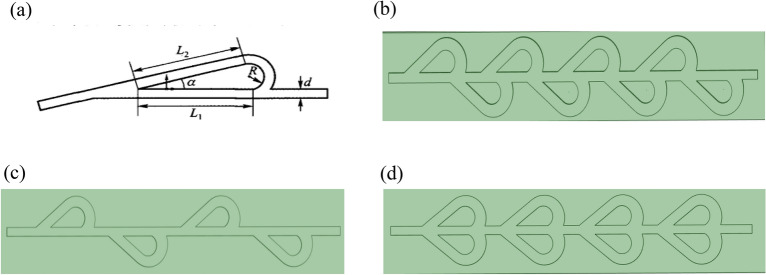
The tesla valve structure of different types: (**a**) the tesla valve structure; (**b**) non-symmetry; (**c**) asymmetries; (**d**) symmetry.

The primary components of the structure consist of shunt length, shunt angle, radius of gyration, and shunt diameter^39^. As shown in Fig. 7.

As illustrated above, the flow of liquid from the right to the left end is known as forward flow, while the flow from the left to the right is known as reverse flow. The bent form inside the valve causes the fluid to flow forward and reverse flow paths have different bending angles and distance traveled, which lead to different losses. Thus,Tesla valve performance is affected by parameters.

#### Parameter analysis and evaluation

The flow of fluid within a tube experiences energy loss and pressure reduction resulting from overcoming internal friction and turbulence generated by momentum exchange when fluid masses collide. The observed phenomenon is that the pressure is different in the fluid flow before and after, that is, the pressure drop. Thus, careful selection and evaluation of valve parameters is necessary. There are various evaluation indexes for Tesla valves, including pressure drop ratio and throttling efficiency^40^.

The pressure drop ratio is expressed by Eq. (9):

9 $$ D_{i} = \frac{{P_{r,i} - P_{r,o} }}{{P_{f,i} - P_{f,o} }} = \frac{{\Delta P_{r} }}{{\Delta P_{f} }} $$

Throttling efficiency^40^ is defined by Eq. (10)

10 $$ E\left( {\Delta P} \right) = \frac{{\left( {Q_{f} - Q_{r} } \right)}}{{Q_{f} }} $$

The pressure drop ratio and throttling efficiency have equivalence, thus, the pressure drop ratio was selected as the evaluation index in the study. In the turbulent flow state, the pressure drop^35^ can be expressed by Eq. (11):

11 $$ \Delta P = \lambda \frac{{\rho \mu^{{2}} L}}{{{2}D}} $$

For Newtonian fluids, the resistance coefficient can be expressed by Eq. (12):

12 $$ \lambda = 0.3116Re^{{{ - }\frac{{1}}{{4}}}} $$

Thus, based on formulas (7), (11) and (12), the expression for pressure drop versus velocity can be obtained by Eq. (13):

13 $$ \Delta P = 0.3116L\rho^{{\frac{3}{{4}}}} V^{{\frac{7}{{4}}}} \mu^{{\frac{1}{{4}}}} D^{{ - \frac{5}{{4}}}} $$

Therefore, the functional relationship can be obtained: $\Delta P$ ~ $V^{{\frac{7}{{4}}}}$.

#### Simulation analysis

Simulation analysis is necessary to determine the optimal parameters for the Tesla valve, considering factors such as valve type, pipe diameter ratio, angle, and valve numbers.

##### Velocity and pressure drop

**Figure 8:**
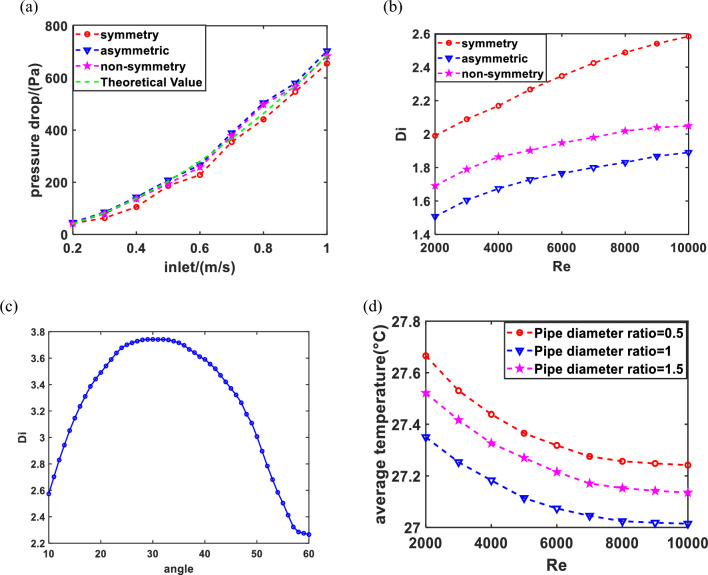
(**a**) Relationship between fluid flow velocity and pressure drop for different valve structures; (**b**) relationship between Re and pressure drop ratio for different valve structures; (**c**) relationship between different tube shunt angles and pressure drop ratio; (**d**) relationship between Re and average temperature for different pipe diameter ratios.

As shown in Fig. 8a, the relationship between fluid flow velocity and pressure drop for different valve structures is analyzed, the results showed that the pressure drop in the symmetrical Tesla valve is smaller than the other two types. This is attributed to the division of flow through the valve into three channels, which subsequently converge into a single flow channel. However, the other valves only have two flow channels, forcing the fluid to circulate through the flow channel repeatedly. Hence, the pressure drop induced by each valve is relatively small, resulting in reduced differential pressure. According to the expression on the P ~ V, which can be used to fit a curve. it can be observed that the three types of Tesla valves exhibit conformity with the theoretical formula.

##### Different types

Figure 8b shows the pressure drop ratio of three different structures at Re numbers ranging from 2000 to 10,000. Although Re is the same, the fully symmetric valve structure has a significantly higher pressure drop ratio than the non-fully symmetric and fully asymmetric valves. The results suggest that the flow channel of the fully symmetric valve demonstrates superior flow characteristics.

##### Different shunt angles

With the increase of shunt angle, the pressure drop progressive increase, which subsequently leads to an improvement in the flow of the pipeline^41^. However, the valve angles excessively wide or small both hinder fluid flow, thereby compromising the overall performance of the pipeline. Consequently, the study establishes a range of diversion angles from 10° to 60° by empirical observations^35^ as shown in Fig. 8c. The pressure drop ratio achieves the maximum value when the angle is 30 degrees, indicating optimal operation of the system. Within the range of 10°–25°, with the increase of shunt angle, the pressure drop ratio increase. whereas the angles from 25° to 35°, the pressure drop ratio remains relatively stable and system performance is achieved optimal. However, the pressure drop ratio decreases once the angle surpasses 35 degrees, reducing system performance.

##### Pipe diameter

The diameter of the manifold pipe significantly affects the fluid flow; if the pipe diameter is too small, results in higher resistance loss in the system, while an excessively large diameter leads to slower flow rates of the manifold fluid. The parameters are typically set at d/D = 0.5, 1, 1.5, and three parameters for the variable to determine the appropriate pipe diameter ratio. The average temperature is used to evaluate the cooling effect of the Tesla valve in a friendly way. The results are shown in Fig. 8d; when the diameter ratio is within a certain range, the cooling effect of the PV is optimal. However, the fluid flow is slow and the cooling effect decreases as the diameter ratio increases. Therefore, the model chooses the diameter ratio of 1 as the optimal cooling parameter selection plan.

##### Valve number

The valve number can be expressed by Eq. (14):

14 $$ N = \left\lfloor {\left( {L_{pv} - 2x} \right)/\left( {l + f_{d} } \right)} \right\rfloor $$

In this research, the photovoltaic plate size is 600 × 90 mm, the pipe diameter is 10 mm and the diversion angle is 30 degrees. The inlet and outlet lengths are 20 mm, the valve length is 40 mm and the valve distance is greater than 20 mm.

**Figure 9:**
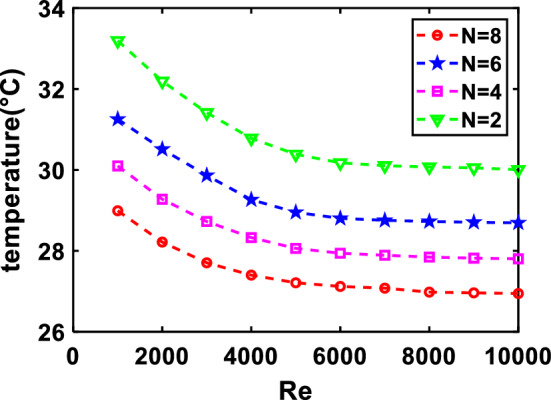
Relationship between Re and temperature for different valve numbers.

Then, the PV average temperature at different Re is plotted in Fig. 9.

By analyzing, the relationship between Re and temperature can be obtained by Eq. (15):

15 $$ {\overline{T}}_{{{\text{cell}}}} = aln\left( {Re} \right) + k $$

Based on theoretical calculations, when the Re = 0, the max temperature should be presented. However, the fluid is stationary in this state; there is still convective heat transfer between the PV and fluid, which results in a decline in temperature. Therefore, the above formula does not consider the case where Re = 0.

According to the above analysis, the function is the logarithmic type and the parameter "a" varies around − 1. While "k" is directly proportional to N, as the number (N) increases, "k" also increases continuously. Simulation results show that the relationship between valve number, Reynolds number and the average PV temperature is deduced by Eq. (16):

16 $$ {\overline{T}}_{{{\text{cell}}}} = - ln\left( {Re} \right) + \frac{40N - 8}{N} $$

According to Eqs. (14) and (16), The average temperature of the panel can be expressed as Eq. (17):

17 $$ {\overline{T}}_{{{\text{cell}}}} = 40 - ln\left( {Re} \right) - \frac{{N_{\max } }}{{\left\lfloor {\left( {L_{pv} - 2x} \right)/\left( {l + fd} \right)} \right\rfloor }} $$

As shown in Fig. 9, increasing the valve numbers while keeping the Re constant results in a better cooling effect on PV temperature. The phenomenon occurs due to the increased contact area between the fluid and the solid, which generates more convective heat transfer. However, the cooling effect does not vary significantly when the Re increases and keeping the valve numbers constant. This observation indicates that the Re affects the cooling effect within a certain range.

In summary, this study analyzes the relationship between pressure drop and velocity is $\Delta P$ ~ $V^{{\frac{7}{{4}}}}$, in which the simulation is verified in three different types. Next, the study further explores the pressure drop ratio of Tesla valves with different structures and the symmetrical Tesla valve with a large pressure drop ratio and good stability is selected. Finally, the parameters that optimize the cooling effect are determined based on simulation analysis, which includes an angle of 30 degrees, a pipe diameter ratio of 1, as many valves as possible and N is taken as 8 in the model.

### Cooling effect in different fluid

Nanofluids are known for excellent heat transfer properties^42^. The cooling effects of four fluids (Water, MgO, TiO2, and Al2O3) in the Tesla valve are analyzed in the study. The parameters shown in Table 3.

**Table 3:** The four fluids parameter.

Type	Density (kg/m^3^)	Specific heat (J/(kg K))	Thermal Conductivity (W/(m K))	Viscosity (N s/m^2^)
Water	998.2	4182	0.6	0.001003
MgO	2900	710	48.4	17.4
TiO_2_	4100	697	8.4	31.2
Al_2_O_3_	3600	773	36	28.2

**Figure 10:**
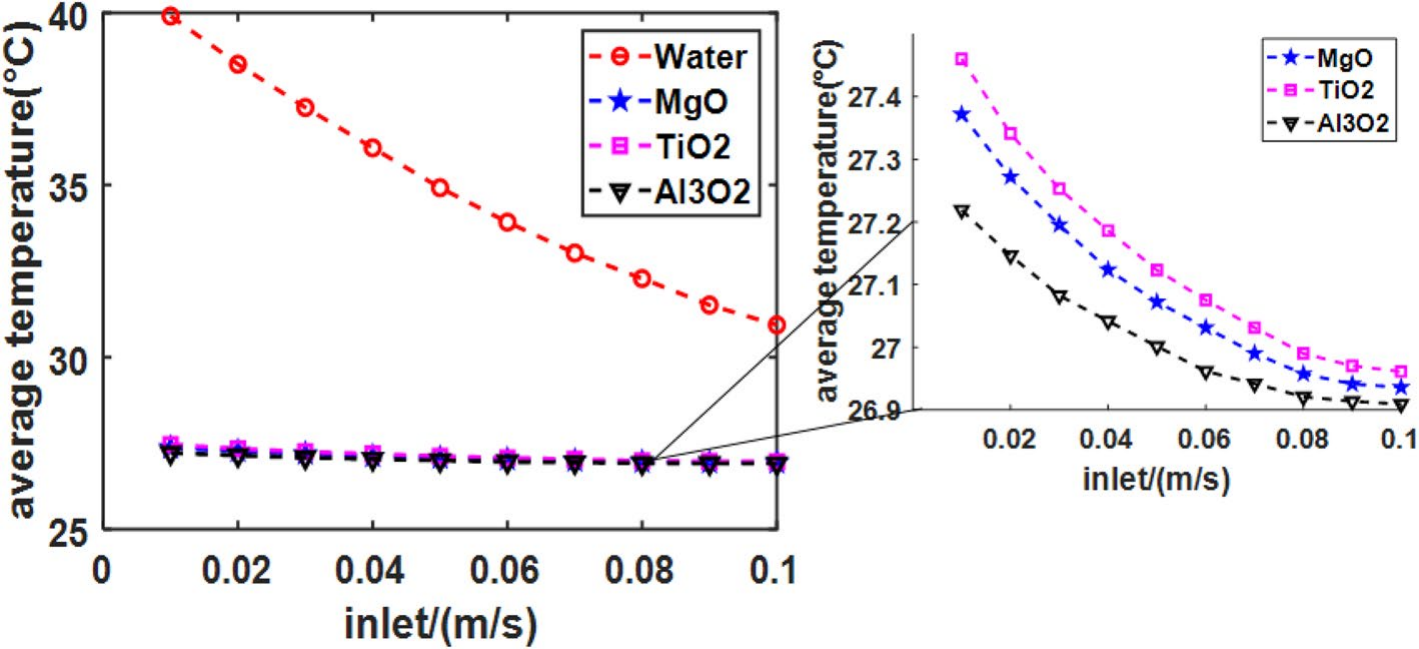
Relationship between inlet fluid flow velocity and average temperature for different fluids.

The results (Fig. 10) indicated that Nanofluids exhibits superior insulation and heat transfer properties as a working fluid. Nanofluids demonstrate better cooling effects than water under the same flow rate conditions. In contrast, nanofluids transfer heat more uniformly than water and result in a better cooling effect even at smaller flow rates. Because the density value and thermal conductivity of nanofluids are very high, which allows them to perform more efficient heat conversion. The temperature drop is smaller for water than nanofluids as a working fluid in the system. Therefore, nanofluids can achieve rapid cooling and heat storage.

## Comparison and discussion

Some comparisons between our conclusion and previous PV/T-related studies are list below, as shown in Table 4.

**Table 4:** Some result comparison about PV/T related study.

References	Method	Electrical efficiency (%)	Thermal efficiency (%)
^43^	PV/T collector using aluminum mini-channels	12.3	57.4
^44^	Using mono and hybrid nanofluid to enhance the performance of a PV/T	11.6	48.2
^45^	filling the PV/T collector with iron filing	16.7	56.2
^46^	PV/T with different tube cross-section patterns	16.9	39.8
Our	PV/T collector with Tesla valve	16.32	59.65

The efficiency is affected by different experimental conditions, such as solar radiation, collector type, environmental conditions, etc. The study chooses similar conditions for comparison as much as possible. The above comparisons show that all the schemes proposed have high thermoelectric efficiency in this study, which is due to the special structure of the Tesla valve flow channel, the positive direction can increase the fluid flow rate and lead to its electrical efficiency and thermal efficiency also reaches a relatively higher level.

## Conclusion

In this paper, the flow channel structure of four different shape structures are compared by simulation analysis, and it is verified that the system with the Tesla valve structure flow channel has superior thermal and electrical efficiency. Several factors affecting the heat dissipation of the Tesla valve are investigated. The main results are presented as follows:

1. the cooling efficiency of the PV/T system with the Tesla valve is superior, electrical efficiency can reach 16.32% and thermal efficiency reach 59.65%.
2. the optimized parameters of the Tesla valve are determined based on simulation analysis, which includes an angle of 30°, a pipe diameter ratio of 1, as many valves as possible and N is taken as 8 in this model, which performs a superior cooling result.
3. Nanofluids have the potential to achieve rapid cooling and heat storage.

In summary, this paper proposes the PV/T with Tesla valve has a significant effect on improving thermoelectric efficiency. The excellent performance of Tesla valve heat dissipation is illustrated, and the PV/T system based on different runner structures and different working conditions can be further investigated in the future, and further experiments can be conducted based on the simulation results. Meanwhile, the Tesla valve structure in this study can also be applied to other heat exchange fields, such as the miniature heat dissipation system of cell phones, aerospace and other fields.

## Data Availability

Data will be made available on request. Ping Zhong (pzhong937@dhu.edu.cn) should be contacted if you want to request the data from this study.
